# Cystic Fibrosis in Egypt

**DOI:** 10.1002/ppul.71899

**Published:** 2026-11-07

**Authors:** Samya Z. Nasr, Salma Fleifil, Eman Fouda, Terez B. Kamel, Heba A. Ali, Maggie Louis Naguib, Mona Mohsen ElAttar, Noussa Ragab Mohamed Elbasha, Osama Taha, Dina T. Sarhan, Deiaaeldin Tamer, Mohammed Ezz El Regal, Rehab Elmeazawy, Nader Fasseeh, Mgr. Malgorzata Libik, Milan Macek

**Affiliations:** ^1^ Michigan Medicine University of Michigan Ann Arbor Michigan USA; ^2^ Ain Shams University Cairo Egypt; ^3^ Cairo University Cairo Egypt; ^4^ Zagazig University Zagazig Egypt; ^5^ Assiut University Assiut Egypt; ^6^ Mansoura University Mansoura Egypt; ^7^ Tanta University Tanta Egypt; ^8^ Alexandria University Alexandria Egypt; ^9^ Department of Biology and Medical Genetics, 2nd Faculty of Medicine and Motol University Hospital Charles University Prague Czechia

**Keywords:** CF in low‐middle‐income countries, CFTR gene variants, CFTR modulator therapy, Cystic Fibrosis, Egyptian people with CF

## Abstract

**Background:**

Published data on cystic fibrosis (CF) among Egyptian people with cystic fibrosis (pwCF) are limited, and most previous reports have been from single centers. This study describes the demographic, clinical, and CFTR variant characteristics of a large multicenter cohort of Egyptian pwCF.

**Methods:**

We conducted a multicenter retrospective descriptive cohort study including pwCF treated at seven Egyptian university children's hospitals from 2020 through 2024. Eligible participants had clinical features consistent with CF and positive sweat chloride testing. Demographic, clinical, sweat chloride, and genetic testing data were collected from medical records. All participants underwent limited CFTR variant testing; participants with one or no identified variants were eligible for additional CFTR sequencing when available.

**Results:**

A total of 475 Egyptian pwCF were included. Participants were recruited from Cairo University, Ain Shams University, Zagazig University, Mansoura University, Tanta University, Alexandria University, and Assiut University. Among the cohort, 175 participants had two identified CFTR variants, 32 had one identified variant, and 268 had no variant identified on available testing. F508del was the most frequent variant, followed by N1303K. Nine novel CFTR variants were identified.

**Conclusion:**

This multicenter retrospective cohort study represents one of the largest reported cohorts of Egyptian pwCF. The findings highlight the genetic heterogeneity of CF in Egypt and support the development of an Egyptian CFTR variant panel, expanded access to comprehensive genetic testing, and broader implementation of newborn screening.

## 1 Introduction

Cystic Fibrosis (CF) is a multisystem genetic disorder characterized by defective chloride ion transport channel and caused by a defect in the CF Transmembrane Conductance Regulator (*CFTR*) gene [1]. The most common variant in Caucasians is a three‐base pair deletion of phenylalanine at nucleotide 508 (F508 del) [1]. Since the *CFTR* gene was identified, over 2000 variants have been reported [2]. CF has not only been diagnosed in the United States and Western Europe but also globally with varying incidence rates [3, 4]. Despite growing awareness about the disease globally, CF remains largely underdiagnosed and uncharacterized, especially in low‐ and middle‐income countries (LMIC) [3, 4].

In some Middle Eastern countries, efforts started to characterize the clinical characteristics and genetics of people with CF (pwCF), particularly in countries with historically low reported incidence of CF [5, 6, 7]. Systematic reviews assessing CF in Arab Countries have demonstrated similar incidence rates among patients of Caucasian and Middle Eastern descent [5, 6]. It is predicted that the F508del is the most common variant in the Middle East, where higher rates of consanguinity contribute to greater rates of homozygosity in pwCF [6]. A recent study described the patterns of CF and its genetic milieu in Bahrain. It estimated an incidence rate of 1 in 9,880 live births and reported three common CF variants, including: p. Gln637HisfsX26 (2043delG), p. His139Leu (H139L), and p. Phe508del, in their population [7].

In Egypt, it was estimated that the incidence of CF is 1:2664 live births, as published in 1993 [8]. Current knowledge of presentation and *CFTR* variant patterns in Egyptian pwCF are limited to a number of single‐center reports [9, 10, 11, 12]. A 2017 study identified 4 novel *CFTR* variants and 13 previously known variants in *CFTR* gene screened in pwCF Egypt [13]. Another study involving 150 Egyptian pwCF found that F508del variant was present in 61.9%, followed by the W1282X variant, which was identified in 25.8%, and the G542X variant in 12.4%. The study noted that patients with a single W1282X variant were at a greater risk of recurrent chest infection and bronchiectasis, and patients with two variants were more likely to have severe symptoms and a more complex treatment course [14]. Similar to Bahrain and other countries in the Middle East, consanguinity rates in this study were reported to be as high as 69% among Egyptian pwCF [14].

The objective of this study was to describe the clinical characteristics, demographic profile, and CFTR variant distribution among Egyptian pwCF treated at seven large university children's hospitals. Characterizing the CFTR variant spectrum in Egypt is important for improving diagnosis, guiding eligibility for CFTR modulator therapy, informing genetic counseling, and supporting development of a national Egyptian CFTR variant panel for newborn screening. This manuscript was prepared in accordance with the STROBE reporting guideline for observational cohort studies.

## 2 Patients and Methods

### 2.1 Study Design and Settings

This was a multicenter retrospective descriptive cohort study of pwCF treated at seven large Egyptian university children's hospitals between January 2020 and December 2024. Participating centers included Cairo University, Ain Shams University, Zagazig University, Mansoura University, Tanta University, Alexandria University, and Assiut University. These hospitals are major pediatric referral centers serving patients from different geographic regions of Egypt.

### 2.2 Participants and Eligibility Criteria

Eligible participants were pwCF who received care at one of the seven participating hospitals during the study period and had clinical features consistent with CF and a positive sweat chloride test (SCT) [15]. Clinical features included failure to thrive (FTT), recurrent pneumonia, bronchiectasis, chronic diarrhea, recurrent sinusitis, liver disease, and/or pancreatic insufficiency. These symptoms were similar to CF symptoms of pwCF in the USA [16], however, FTT, respiratory symptoms, and liver disease were worse than reported in the USA [9]. Participants were included if de‐identified demographic, clinical, SCT, and genetic testing data were available. Duplicate records across centers were reviewed and removed when identified. Participants were excluded if SCT was negative or unavailable, or if the available clinical information was insufficient to confirm eligibility.

### 2.3 Diagnostic Criteria

SCT was performed using the Macroduct® Sweat Collection System or equivalent systems available at participating centers. A positive SCT was defined according to published CF Foundation diagnostic guidelines [15]. Where available, sweat chloride values were recorded as mmol/L. A sweat chloride concentration ≥ 60 mmol/L was considered positive.

### 2.4 Sample Size

The study size was determined by the number of eligible pwCF treated at the seven participating centers during the study period. No formal sample size calculation was performed because the study was descriptive and included all available eligible participants.

### 2.5 Variables

The variables collected included age, sex, treating center, SCT, consanguinity, body mass index percentile (BMI), FEV1 percent predicted (ppFEV1); the ppFEV1 measurements were obtained according to ATS/ERS standards in pwCF 6 years and older, pancreatic insufficiency, liver disease, bronchiectasis, recurrent pneumonia, hemoptysis, pneumothorax, CF‐related diabetes, hospitalization history, and CFTR variants. Genetic variables included number of identified CFTR variants, zygosity, variant class, HGVS nomenclature, legacy nomenclature, and pathogenicity classification.

### 2.6 Genetic Testing Methods

All 475 participants underwent initial limited CFTR variant testing in Egypt using a reverse hybridization StripAssay covering 34 CFTR variants and one polymorphism. A total of 450 participants had one or no variant identified on initial testing and were eligible for additional sequencing. Of these, 150 underwent additional sequencing, while 300 did not because of restrictions on sending samples outside Egypt following the COVID‐19 pandemic, financial limitations, or sample unavailability. The samples were sent prior to 2020. The samples sent for full sequencing were 10 samples from the 1 variant and 140 from the non‐variant groups. Two variants were identified in all the samples sent. (Diagnostic Workflow chart)

Participants with one or no identified CFTR variant were eligible for additional testing of the CFTR coding region and selected intronic regions when export of samples and resources permitted. Additional sequencing was performed at the Department of Biology and Medical Genetics, 2nd Faculty of Medicine and Motol University Hospital, Charles University, Prague, Czechia.

### 2.7 Sample Collections

For the limited variants analysis that was done in Egypt, the test was done by the StripAssay reverse hybridization technique. The assay covers 34 *CFTR* variants and 1 polymorphism in the *CFTR* gene. (ViennaLab Diagnostics. StripAssay® CFTR (IVS8 PolyT, 34 mutations): Product information and technical manual). The variants tested have been recommended by the American College of Medical Genetics [17]. If two likely pathogenic *CFTR* variants were identified per CFTR1 and CFTR2, that was recorded. If one variant or no variants were identified, the patients' samples were sent to the Department of Biology and Medical Genetics, 2nd Faculty of Medicine and Motol University Hospital, Charles University, Prague, Czechia, for further analysis. The genetic testing for pwCF that was done at Charles University includes the procedure below.

Genomic DNA was isolated from dry blood spots using the **MagCore Genomic DNA Tissue Kit**. We have utilized a ‘cascade approach’ by initially testing for the most common mutations using the panel of the 50 most common *CFTR* variants in the European‐derived populations Elucigene CFEU v2 (Elucigene, UK), followed by MPS‐based analysis of the entire *CFTR* coding region, adjacent splice site junctions, and several introns using a locus‐specific library preparation assay (*CFTR* NGS assay™; Devyser, Sweden; www.devyser.com). MPS sequencing was performed on the MiSeq System™ (Illumina, USA; www.Illumina.com). Bioinformatic analysis was carried out using the SOPHiA Platform for Hereditary Disorders™ online software (www.sophiagenetics.com). Where applicable, positive cases were confirmed by targeted Sanger DNA sequencing on ABI 3130xl DNA Analyzer™ (ThermoFisher, USA; www.thermofisher.com). The linkage phase of detected mutations was established by testing fewer common mutations and using IGV software (Broad Institute). For the *CFTR* variant nomenclature, we used the recommendation of the Cystic Fibrosis Mutation Database (*CFTR1*; Toronto, Canada; www.gene.Sickkids.on.ca). However, in particular in the Discussion section we resorted to the still more widely understood ‘legacy nomenclature’ indicated in parentheses. Variant pathogenicity was assessed according to the ‘Clinical and Functional Translation of *CFTR*’ database (*CFTR2*; Baltimore, USA; www.cftr2.org) where applicable.

The study population was reported as 3 groups; pwCF with 2 identified *CFTR* variants, pwCF with 1 identified *CFTR* variant, and pwCF that genetic testing was negative for the 34 CFTR variants and 1 polymorphism that was available in Egypt.

### 2.8 Bias

Several potential sources of bias were considered. Because participating hospitals are tertiary referral centers, the cohort may overrepresent individuals with more severe disease or those with access to specialized care. To reduce selection bias, all eligible pwCF treated at participating centers during the study period were included when data were available. Information bias was possible because clinical variables were abstracted retrospectively from medical records and may not have been recorded uniformly across centers. Genetic ascertainment bias was also possible because full CFTR sequencing was not available for all participants with one or no variant identified on limited testing.

### 2.9 Missing Data

Missing data were expected because of the retrospective design. The denominator for each variable is reported in the tables. Variables with substantial missingness, including pancreatic insufficiency, were interpreted cautiously.

### 2.10 Statistical Analysis

Descriptive statistics were used to summarize demographic, clinical, and genetic characteristics. Continuous variables were summarized using mean and standard deviation. Categorical variables were summarized using frequencies and percentages. CFTR variant frequencies were calculated at the allele level and, where appropriate, at the patient level. Participants were grouped according to the number of identified CFTR variants: two variants, one variant, and no variant identified on available testing. Missing data were reported for each variable, and analyses were conducted using available data without imputation. Comparisons among the three genetic‐testing groups were performed in descriptive statistical analysis.

This study is reported in accordance with the Strengthening the Reporting of Observational Studies in Epidemiology — STROBE — guideline for observational cohort studies. The completed STROBE checklist is provided as Table S1.

## 3 Results

During the study period, 475 eligible pwCF were identified and included in the analysis. All had clinical features consistent with CF and positive SCT. All 475 underwent limited CFTR variant testing. Based on available genetic testing followed by full sequencing of 150 pwCF for some that have 1 or no variant identified after the limited testing; 175 participants had two identified CFTR variants, 32 had one identified variant, and 268 had no variant identified. Reasons for lack of full sequencing of pwCF that have 1 or no variants included restrictions on exporting samples following the COVID‐19 pandemic, financial limitations, and/or sample unavailability. Nine novel variants were identified, defined as variants not previously reported in the CFTR2, CFTR‐France, or ClinVar databases at the time of analysis (all accessed on January 5, 2025): p.Lys381Aspfs*5, p.Asp1214Ilefs*14, p.Glu632Thrfs*9, p.Glu632Thrfs*9, *p.(Ser912Cysfs*11), CFTRdele11‐15, G178X, p.(Gly461Val), 1‐16_19‐24dup and c.1404_1408del p.(Met469AspfsTer12). The age range was 1.85–18.23 years of age which was notable for generally young age. Historically, the median age at diagnosis has been 3–4 years of age. However, recent data from the participating centers report a median diagnostic age of 6–8 months. Males were slightly higher than females, with males at 62.5% of the study population. The 3 groups showed nearly similar SCT mean values, rate of consanguinity, and BMI percentile. Unfortunately, the rates of pancreatic insufficiency and consanguinity were partially reported. Complications and hospitalizations appeared more frequent in the group with two identified variants, and pwCF were younger than in the other two groups. Pathogenic variants were identified from CFTR2 website and Burgel et al. [18, 19]. There was wide variation in FEV1% pp (37.08–96.26). Children tested were 6 years and older; the wide range of FEV1% pp is an indication of the wide variation of the disease in the Egyptian pwCF. Mean BMI percentile was low in the three groups (range 12.69%–17.14%). (Table 1).

**Table 1:** Patient demographics and characteristics based on number of identified mutations.

	2 Variants (*n* = 175)		One Variant (*n* = 32)		No Variant Identified (*n* = 268)	
Variable (mean SD)	Means ± SD	%	Means ± SD	%	Means ± SD	%
Age (y) M ± SD	6.75 ± 4.9		11.13 ± 7.10		8.18 ± 4.80	
Male (*n*)	102	58.29	20	62.50	149	55.60
Sweat chloride (mml/l)	84.7 ± 24.2		81.4 ± 32.39		87.23 ± 26.2	
Consanguinity (*n*)	31/49	63.27	6/9	66.67	22/32	68.75
pancreatic Insufficiency (*n*)	48		0		70/77	90.9
FEV1% PP	63.32 ± 19.9	Range: 43.42–83.22	66.16 ± 30.10	Range: 36.06–96.26	62.68 ± 25.60	Range: 37.08–88.28
BMI percentil	15.34 ± 1.8	Range: 13.54–16.98	15.09 ± 2.40	Range: 12.69–17.49	15.26 ± 1.60	Range: 13.66–16.86

Table 2 outlines the most common genetic variants within the Egyptian pwCF. Supplement Table includes all variants tested. It lists the most common variants Legacy name, *CFTR* variant classes, Human Genome Variation Society nomenclature (HGVS) cDNA, HGVS protein, and clinical variants (clin var) new nomenclature. Among participants with identified CFTR variants, F508del (c.1521_1523delCTT) accounted for 156 alleles, or 40.83%, (sixty‐three patients classified as homozygous and thirty as heterozygous for F508del), it is classified as Class II [17, 18]. That was followed by N1303K (c.3909 C > G), also Class II variant, appeared in 4.95% of alleles tested, with 8 homozygous and 3 heterozygous instances. That was followed by G542X (c.1624 G > T) a Class I variant and present in 4.17% of the alleles and included 8 homozygous carriers. There were 9 novel CFTR variants reported. Figure 1 shows the frequency and distribution of most common genetic variants among Egyptian pwCF. The most common variants were class II, and class I variants were the least common. Most variants were homozygous.

**Table 2:** The most common gene variants among CF patient cohort.

Mutation Legacy name and/or protein change	New Nomenclature c. DNA	Mutation Class	Allele (n)	allele%	Number of patients homozygous for the allele	Number of patients heterozygous for the allele
F508del	c.1521_1523delCTT	Class II	156	40.83	63	30
N1303K	c.3909 C > G	Class II	24	4.95	8	3
G542X	c.1624 G > T	Class I	16	4.17	8	
S546R	c.1647 T > G	Class III	13	3.39	6	1
2183AA > G	c.2051_2052delAAinsG	Class I	13	3.39	6	1
R334W	c.1000 C > T	Class IV	9	2.34	3	3
3120 + 1 G > A	c.2988 + 1 G > A	Class I	6	1.56	3	
3670delA	c.3540del	Class I	5	1.30	3	_
3272‐26 A	c.3140−26 A > G	Class V	8	2.08		5
G461V	c.1382 G > T	Class III novel	4	1.04	2	1
p. Met469AspfsT*r11	c.1404_1408del	Class I‐ Novel	4	1.04	2	
2789 + 5 G > A	c.2789 + 5 G > A	Class V	4	1.04	2	
R1066H	c.3197 G > A	Class IV	4	1.04	2	
W1282X	c.3846 G > A	Class I	4	1.04	2	1

**Figure 1:**
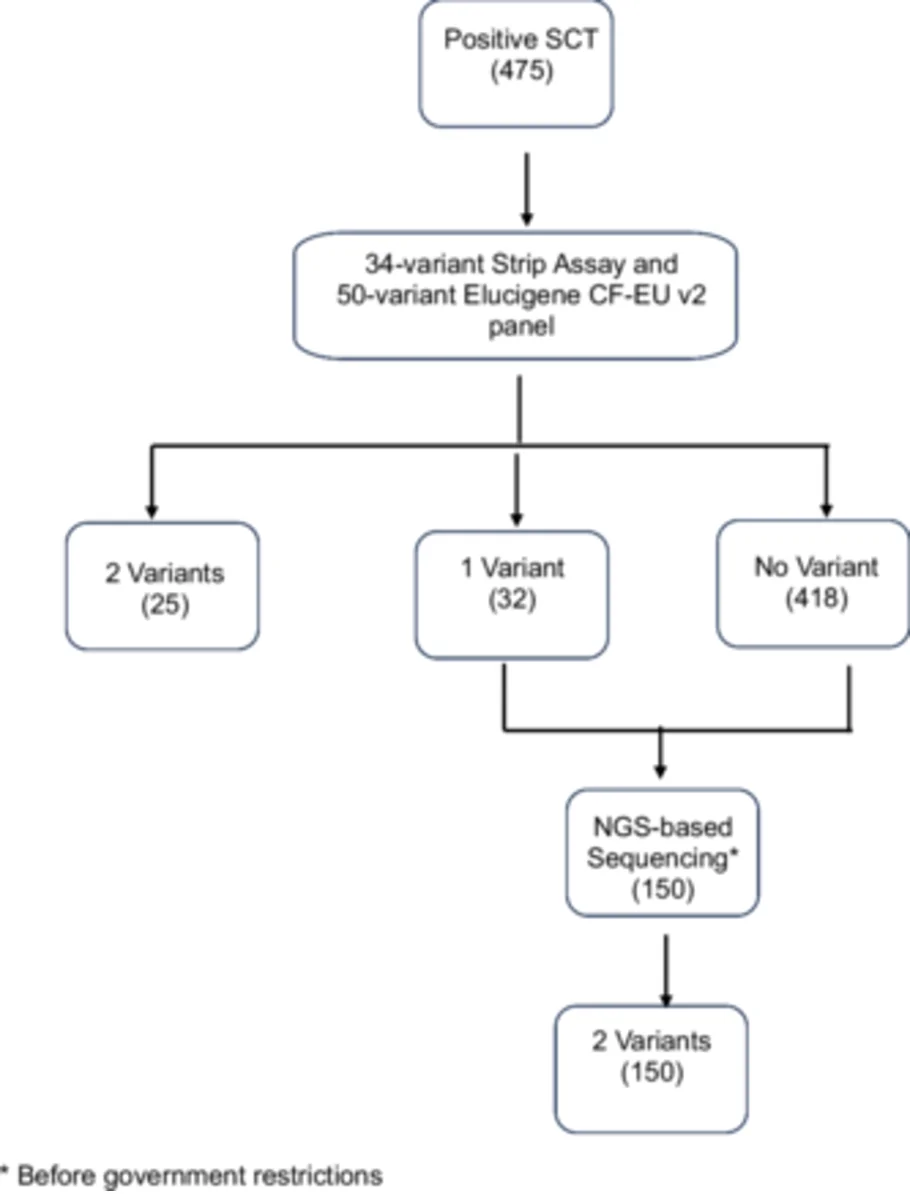
Frequency and distribution of most common genetic variants among Egyptian pwCF. [Color figure can be viewed at wileyonlinelibrary.com]

## 4 Discussions

This study presents the largest multi‐center Egyptian pwCF cohort to date. This is in comparison to prior reports from Egypt and neighboring regions [5, 6, 7, 8, 9, 10, 11, 12, 13, 14]. The study is a nationwide assessment of 475 Egyptian pwCF, including *CFTR* genotyping in all individuals; 207 pwCF either have 1 or 2 variants identified, making it one of the largest CF cohorts reported from the Middle East. By analyzing data from seven major pediatric centers across Egypt, the study offers novel insights into the genetic and clinical landscape of CF in a LMIC, with important implications for diagnosis, treatment strategies, newborn screening implementation, and health policy development. The most frequent *CFTR* mutation identified was the F508del variant, consistent with its global predominance, particularly among Caucasian populations. This variant accounted for 40.83% of the alleles in our cohort, supporting earlier Egyptian studies and regional data from Middle Eastern populations, such as in Bahrain and Saudi Arabia, where F508del similarly predominates [7, 9, 10, 11, 12]. This also aligns with global and regional findings [3]. To explain the high prevalence of the F508del mutation in Egypt, several factors could have contributed to that, including historical Genetic Drift and Founder's Effect that occurred over time. Populations that were isolated or had a small initial population size might have experienced a founder effect, where certain genetic traits, such as the F508del mutation, became fixed or more common in the population [19, 20, 21].

Another contributing factor is Egypt's complex genetic admixture, shaped by its geographic location and historical role as a hub for trade, conquest, and migration. This has likely influenced the distribution of *CFTR* mutations, including those commonly seen in European, Middle Eastern, and North African populations. Additionally, the relatively high frequency of the F508del variant in Egypt may be attributed to historical gene flow from Europe, where this variant is highly prevalent. However, the relatively high frequency of other variants, including N1303K, G542X, R334W, and several frameshift or splicing mutations, and the presence of less frequent variants (e.g., R1066H, M469fs, 3120 + 1 G > A) highlight the genetic heterogeneity in Egypt, potentially due to factors listed above. This diversity highlights the limitations of using European‐derived CFTR screening panels and emphasizes the need for a tailored, population‐specific variant panel to improve diagnostic accuracy.

A notable finding in our study is the remarkably high rate of consanguinity (63.27%–68.75%) among affected individuals, more than double the estimated national average of 35% [14]. However, it was only reported in 90 of 475 pwCF (19% of the total). High consanguinity rates could be associated with increased homozygosity. However, the generalizability of a rate derived from such a small, non‐random subsample is limited.

Clinically, even though the reported pancreatic insufficiency in this study was limited, other studies from Egypt showed that it is prevalent at a rate higher than 90% [9, 10, 22]. That was higher than the rates in the United States (~85%) [15]. This may be attributable to the variant profile observed, particularly the high prevalence of severe, class I–III mutations. Notably, the subgroup of pwCF with two identified mutations was younger and experienced a higher frequency of complications, like pneumothorax, hemoptysis, CF‐related diabetes, and hospitalizations. These findings suggest that earlier diagnosis correlates with increased clinical vigilance and perhaps earlier disease management since this group was diagnosed at a younger age than the other two groups.

Encouragingly, the median age at diagnosis for Egyptian pwCF has been significantly improved. While historical data indicated diagnosis typically occurred between 3 and 4 years of age, recent data from the participating centers report a median diagnostic age of 6–8 months. This improvement likely reflects increased physician awareness, broader availability of SCT, and the availability of CF medications. All pwCF had positive SCT (≥ 60 mmol/L) and had phenotypic characteristics of CF. They all underwent limited‐panel testing in Egypt. Nevertheless, over half of the cohort (268/475) did not undergo full genetic sequencing testing (after a negative mutation panel), primarily due to financial and resource constraints. This highlights a critical gap in comprehensive CF care and underscores the need for national investment in genetic testing infrastructure. Guo et al. discussed gaps in global access to genetic diagnostics [4].

Given the transformative impact of the highly effective CFTR modulator therapies on CF management, and the recent availability of Elexacaftor/Tezacaftor/Ivacaftor (ETI) in Egypt through a donation program from Vertex Pharmaceuticals ^inc^, our findings can carry important clinical implications. Several identified mutations in this cohort—including F508del and N1303K—are eligible for ETI therapy. However, the absence of full genetic testing in over half of the studied Egyptian pwCF (268/475) presents a major barrier to accessing these targeted therapies. Genotyping is therefore critical to ensure equitable access to modulator therapy and guide individualized treatment.

Furthermore, identifying the full mutation spectrum in pwCF in Egypt enables the implementation of CF NBS across the country which is now a pilot program and will be implemented soon across the country, which will help accurately identifying the prevalence of the disease there.

## 5 Limitations

This study has several limitations. First, because participants were recruited from tertiary university hospitals, the cohort may not fully represent all Egyptian pwCF, particularly those who remain undiagnosed or lack access to specialty care. Second, the retrospective design limits the completeness and uniformity of clinical data across centers. Some variables, including pancreatic insufficiency, were incompletely reported. Third, full CFTR sequencing was not available for all participants, especially those with one or no variant identified on initial limited testing. As a result, the frequency of rare or novel variants may be underestimated. Fourth, because the study was descriptive, associations between genotype and clinical outcomes should be interpreted cautiously. Finally, the study does not provide a population‐based estimate of cystic fibrosis incidence or prevalence in Egypt. Despite these limitations, the study includes patients from seven major referral centers across different regions of Egypt and provides the most comprehensive multicenter description to date of clinical and CFTR variant characteristics among Egyptian people with cystic fibrosis. The findings are most generalizable to Egyptian children and adolescents receiving care at tertiary pediatric centers and may not capture milder, undiagnosed, or adult cases.

In conclusion, this study is, to our knowledge, the largest multicenter study of Egyptian pwCF and among the largest reported cohorts from the Middle East. It provides valuable data for *CFTR* variant distribution and clinical characteristics of these pwCF. Limited access to full genetic testing remains a major barrier to precision treatment. These findings support the development of a national Egyptian *CFTR* variant panel and broader implementation of newborn screening across the country.

## Author Contributions

**Samya Z. Nasr:** conceptualization, formal analysis, visualization, methodology, investigation, supervision, project administration, funding acquisition, resources, data curation, writing – review and editing, validation, writing – original draft. **Salma Fleifil:** data curation, software, validation, writing – review and editing, formal analysis, writing – original draft. **Eman Fouda:** writing – review and editing, supervision, investigation, data curation, formal analysis, resources. **Terez B. Kamel:** conceptualization, data curation, validation, resources, supervision, writing – review and editing. **Heba A. Ali:** data curation, validation, resources, writing – review and editing, investigation. **Maggie Louis Naguib:** conceptualization, data curation, writing – review and editing, supervision, validation, investigation, formal analysis. **Mona Mohsen ElAttar:** methodology, data curation, writing – review and editing, validation. **Noussa Ragab Mohamed Elbasha:** writing – review and editing, data curation, validation, investigation. **Osama Taha:** data curation, formal analysis, writing – review and editing, resources, validation, investigation. **Dina T. Sarhan:** resources, validation, writing – review and editing, investigation, data curation. **Deiaaeldin Tamer:** validation, resources, investigation, writing – review and editing, data curation, formal analysis. **Mohammed Ezz El Regal:** validation, resources, writing – review and editing, investigation, data curation, formal analysis. **Rehab Elmeazawy:** validation, resources, investigation, data curation, formal analysis, writing – review and editing. **Nader Fasseeh:** writing – review and editing, data curation, investigation, validation, resources. **Mgr Malgorzata Libik:** software, validation, resources, investigation, methodology, writing – review and editing, formal analysis, writing – original draft. **Milan Macek Jr:** conceptualization, formal analysis, visualization, writing – review and editing, writing – original draft, investigation, validation, funding acquisition.

## Ethics statement

Ethical approval was obtained from the institutional review boards or ethics committees of each participating Egyptian university and from the University of Michigan Institutional Review Board (HUM00264457). The requirement for informed consent was waived because the study was retrospective and used de‐identified data. Data were shared and analyzed in de‐identified form to protect patient privacy.

## Conflicts of Interest

The authors declare no conflicts of interest.

## Supporting information


Supporting File

## Data Availability

The data that support the findings of this study are available on request from the corresponding author. The data are not publicly available due to privacy or ethical restrictions.
