# Supplementary material for: Cystic Fibrosis in Egypt
Source: Pediatr Pulmonol. 2026 Nov 7;61(11):e71899. doi: 10.1002/ppul.71899 (PMC13744004; doi:10.1002/ppul.71899)
Supplement: Supplementary file 1 [file PPUL-61-0-s001.docx]

Supplemental Table. Frequency of the most common gene variants among pwCF cohort of all variants tested.

| **Variants (Legacy name)** | **CFTR Functional Class** | **Burgel Clinical Class ^17^** | **HGVS (cDNA/Protein)^1^** | **HGVS protein** | **Clinical Variant Accession (CVC)^2^** | **CFTR2 classification** | **Allele count** |
| --- | --- | --- | --- | --- | --- | --- | --- |
| F508del | Class II |  | c.1521_1523delCTT | p.Phe508del | VCV000007105 | CF-causing variant | 156 |
| N1303K | Class II |  | c.3909C>G | p.Asn1303Lys | VCV000007136 | CF-causing variant | 19 |
| CFTRdele17a-18 | Class I | true class I | c.(2988+1_2989-1)_(3468+1_3469-1)del | CFTRdele19-21 | VCV001705983 | CF-causing variant | 15 |
| S549R(T>G) | Class II |  | c.1647T>G | p.Ser549Arg | VCV000040190 | CF-causing variant | 15 |
| 2183delAA>~~ins~~G | Class I | true class I | c.2051_2052delAAinsG | p.Lys684Serfs*38 | VCV000035837 | CF-causing variant | 13 |
| G542X | Class I | true class I | c.1624G>T | p.Gly542Ter | VCV000007115 | CF-causing variant | 16 |
| R1162X | Class I | true class I | c.3484C>T | p.Arg1162Ter | VCV000007137 | CF-causing variant | 6 |
| 4382delA | Class I | probably responsive to ETI | c.4251delA | p.Glu1418ArgfsX14 | VCV000053933 | CF-causing variant | 6 |
| F508del_L467F | Unknown Class |  | c.1521_1523delCTT_c.1399C>T | p.Phe508del_p.Leu467Pro |  | revertant complex allele | 5 |
| R334W | Class IV |  | c.1000C>T | p.Arg334Trp | VCV000514262 | CF-causing variant | 9 |
| 1548delG | Class I | true class I | c.1418delG | p.Gly473GlufsX54 | VCV000053251 | CF-causing variant | 5 |
| 1898+3A>C | Class V |  | c.1766+3A>C | n/a | VCV000053378 | n/a | 4 |
| 2789+5G>A | Class V |  | c.2657+5G>A | n/a | VCV000038497 | CF-causing variant | 4 |
| 3129del4 | Class I | true class I | c.2997_3000del | p.Ile1000Ter | VCV000053618 | CF-causing variant | 4 |
| CFTRdele20 | Class I |  | c.(3717+1_3718-1)_(3873+1_3874-1)del | n/a | n/a | CF-causing variant | 4 |
| G178X | Unknown Class |  | c.532G>T | p.Gly178Ter | n/a | CF-causing variant | 4 |
| M152V | Unknown Class |  | c.454A>G | p.Met152Val | VCV000053955 | n/a | 4 |
| n/a | Unknown Class |  | c.1405A>G | p.(Met469Aspfs*12) | n/a | NOVEL | 4 |
| 3670delA | Class I |  | c.3540delA | p.Lys1180AsnfsX12 | n/a | n/a | 6 |
| G178R | Unknown Class |  | c.532G>A | p.Gly178Arg |  | CF-causing variant | 3 |
| Q378X | Unknown Class | true class I | c.1132C>T | p.Gln378Ter |  | CF-causing variant | 3 |
| 1525-1G>A | Class V | true class I | c.1393-1G>A | n/a | VCV000053242 | CF-causing variant | 3 |
| A141D | Unknown Class |  | c.422C>A | p.Ala141Asp | n/a | n/a | 3 |
| CFTRdele2ins182 | Class I | true class I | c.(53+1_54-1)_(164+1_165-1)del | CFTRdele2 | VCV001705984 | CF-causing variant | 3 |
| G970S | Unknown Class |  | c.2908G>A | p.Gly970Ser | VCV000053589 | n/a | 3 |
| n/a | Class I |  | c.1141_1142delAA | p.Lys381Aspfs*5 |  | NOVEL | 3 |
| 3199del6 | Class I | true class I | c.3067_3072delATAGTG | p.Ile1023_Val1024del | VCV000038480 | CF-causing variant | 1 |
| 3130delA | Class I | true class I | c.2998delA | p.Ile1000LeufsX2 |  | n/a | 2 |
| CFTRdele11-15 | Class I |  | c.(1584+1_1585-1)_(2908+1_2909-1)del | n/a |  | NOVEL | 2 |
| D579G | Unknown Class |  | c.1736A>G | p.Asp579Gly |  | VCC | 2 |
| G461V | Unknown Class |  | c.1381GT | p.(Gly461Val) |  | NOVEL | 5 |
| R1066C | Class II |  | c.3196C>T | p.Arg1066Cys | VCV000007162 | CF-causing variant | 2 |
| R1066H | Class IV |  | c.3197G>A | p.Arg1066His | VCV000007158 | CF-causing variant | 4 |
| 2221insA | Class I | true class I | c.2089dup | p.Arg697LysfsX33 | VCV000053439 | CF-causing variant | 2 |
| 444delA | Class I | true class I | c.313delA | p.Ile105SerfsX2 | VCV000048684 | CF-causing variant | 2 |
| L867X | Unknown Class | true class I | c.2600T>A | p.Leu867Ter | VCV000694035 | n/a | 2 |
| R75X | Class I | true class I | c.223C>T | p.Arg75Ter | VCV000048672 | CF-causing variant | 2 |
| R785X | Class I | true class I | c.2353C>T | p.Arg785Ter | VCV000053480 | CF-causing variant | 2 |
| W1282X | Class I |  | c.3846G>A | p.Trp1282Ter | VCV000007121 | CF-causing variant | 5 |
| 1717-1G>A | Class V | true class I | c.1585-1G>A | n/a | VCV000007112 | CF-causing variant | 1 |
| 2043delG | Class I | true class I | c.1911delG | p.Gln637HisfsX26 | RCV000029489.23 | CF-causing variant | 1 |
| 3120+1G>A | Class I | true class I | c.2988+1G>A | n/a | VCV000007224 | CF-causing variant | 6 |
| 3272-26A>G | Class V |  | c.3140-26A>G | n/a | VCV000035864 | CF-causing variant | 5 |
| 541del | Class I | true class I | c.409_412del | p.Leu137TyrfsX15 | VCV000053886 | CF-causing variant | 1 |
| E1104X | Class I | true class I | c.3310G>T | p.Glu1104Ter | VCV000053715 | CF-causing variant | 1 |
| G1244R | Unknown Class |  | c.3730G>A | p.Gly1244Arg | VCV000053796 | n/a | 1 |
| 3199del6_I148T | Class I + Unknown Class |  | c.443T>C_c.3067_3072delATAGTG | p.Ile148Thr_p.Ile1023_Val1024del | n/a | complex allele | 1 |
| n/a | Class I |  | c.3639del | p.Asp1214IlefsX14 | n/a | n/a | 1 |
| n/a | Class I |  | c.1894_1895delGA | p.Glu632Thrfs*9 |  | n/a | 1 |
| Q1012X | Unknown Class | true class I | c.3034C>T | p.Gln1012Ter | n/a | CF-causing variant | 1 |
| n/a | Class I |  | c.4053_4054delinsAG | p.Gln1352Glu | n/a | NOVEL | 1 |
| R553X | Class I | true class I | c.1657C>T | p.Arg553Ter | VCV000007122 | CF-causing variant | 1 |
| S431X | Class I |  | c.1291del | p.(Ser431Valfs*11) | n/a | Novel | 1 |
| W79X | Unknown Class | true class I | c.236G>A | p.Trp79Ter | n/a | CF-causing variant | 1 |
| n/a | Unknown Class |  | c.(?_1_2909-1)_(3717+1_*1_?)dup | n/a | n/a | Novel | 1 |
| 1949del84 | Unknown Class |  | c.1820_1903del84 | p.Met607_Gln634del | VCV000053396 | CF-causing variant | 1 |
| 4010del4 | Class I | true class I | c.3882_3885delTATT | p.Ile1295PhefsX32 | VCV000053838 | CF-causing variant | 1 |
| 875+1G>A | Class I | considered to be responders to ETI. | c.743+1G>A | n/a | VCV000054051 | CF-causing variant | 1 |
| A120T | Unknown Class |  | c.358G>A | p.Ala120Thr | VCV000053774 | VCC | 1 |
| CFTRdele17b,18 | Class I | true class I | c.(3139+1_3140-1)_(3468+1_3469-1)del | n/a | n/a | CF-causing variant | 1 |
| D1445N | Unknown Class |  | c.4333G>A | p.Asp1445Asn | VCV000053941 | n/a | 1 |
| G673X | Unknown Class | true class I | c.2017G>T | p.Gly673Ter | VCV000053941 | CF-causing variant | 1 |
| G85E | Class II | true class I | c.254G>A | p.Gly85Glu | VCV000007143 | CF-causing variant | 1 |
| n/a | Class I | true class I | c.147_150delATCT | p.Ser50LysfsX40 | VCV000618923 | CF-causing variant | 1 |
| n/a | Class I |  | c.2735del | p.(Ser912Cysfs*11) | n/a | Novel | 1 |
| n/a | Class I | true class I | c.1190dupT | p.Thr398AsnfsX13 | n/a | CF-causing variant | 1 |
| R248K | Unknown Class |  | c.743G>A VUS | p.Arg248Lys | n/a | n/a | 1 |
| R334L | Unknown Class |  | c.1001G>T | p.Arg334Leu | VCV000053160 | CF-causing variant | 1 |
| R668C | Unknown Class |  | c.2002C>T | p.Arg668Cys | VCV000035835 | non-pathogenic | 1 |
| S466X_R1070Q | Unknown Class |  | c.[1397C>G;3209G>A] | p.[Ser466X;Arg1070Gln] | RCV000785639 | complex allele | 1 |
| S977F | Unknown Class |  | c.2930C>T | p.Ser977Phe | VCV000053601 | CF-causing variant | 1 |
| Y849X | Unknown Class | true class I | c.2547C>A | p.Tyr849Ter | VCV000053509 | CF-causing variant | 1 |
| p.(Met469AspfsTer12) | Class I |  | c.1404_1408del |  |  | Novel | 4 |

^1^VUS - Variant of unknown significance

^2^VCV - Variant with Varying Clinical Consequences by CFTR2 database
